# Detection and prevalence of depression among adult type 2 diabetes mellitus patients attending non-communicable diseases clinics in Lilongwe, Malawi

**DOI:** 10.1186/s13033-020-00413-3

**Published:** 2020-11-04

**Authors:** Michael Udedi, Brian W. Pence, Robert C. Stewart, Adamson S. Muula

**Affiliations:** 1 Department of Mental Health, College of Medicine, University of Malawi, Chichiri, P/Bag 360, Blantyre 3, Malawi; 2 Department of Clinical Services, Ministry of Health, P. O. Box 30377, Capital City, Lilongwe 3, Malawi; 3 Department of Public Health, College of Medicine, University of Malawi, Chichiri, P/Bag 360, Blantyre 3, Malawi; 4 Africa Center of Excellence in Public Health and Herbal Medicine, College of Medicine, University of Malawi, Chichiri, P/Bag 360, Blantyre 3, Malawi; 5 Epidemiology Department, University of North Carolina At Chapel Hill Gillings School of Global Public Health, 135 Dauer Dr, Chapel Hill, NC 27599 USA; 6 Division of Psychiatry, University of Edinburgh, Royal Edinburgh Hospital, Morningside Park, Edinburgh, EH10 5HF UK; 7 Malawi Epidemiology and Intervention Research Unit (MEIRU), Box 148, Lilongwe 3, Malawi

**Keywords:** Depression, Type 2 diabetes mellitus, Malawi

## Abstract

**Background:**

Depression is associated with chronic physical illnesses and negatively affects health outcomes. However, it often goes undiagnosed and untreated. We investigated the prevalence of depression among adult type 2 diabetes mellitus (T2DM) patients attending non-communicable diseases (NCD) clinics in Lilongwe, Malawi, and estimated the level of routine detection by NCD clinicians. This study set out to determine the prevalence of major depression and its detection among adult type 2 diabetes mellitus (T2DM) patients attending NCD clinics in Lilongwe, Malawi.

**Methods:**

In a cross-sectional study design, 323 T2DM patients aged ≥ 18 years were screened for depression with the Patient Health Questionnare-9 (PHQ-9) followed by diagnostic assessment with the Structured Clinical Interview for DSM-IV (SCID). We analysed the association between presence of major depression and sociodemographic factors using logistic regression.

**Results:**

Three quarters of the participants (76%) were females. The participants’ ages ranged from 21–79 years. Of the 323 participants, 58 (18%) met criteria for DSM-IV major depression. None of the cases of major depression had been identified by the NCD clinicians. Major depression was found not to be significantly associated with any of the sociodemographic factors.

**Conclusions:**

We found that depression is common among NCD clinic attendees with T2DM in Malawi, and poorly detected by NCD clinicians. Given the high prevalence and challenges in clinical identification, integration of depression screening with a standardized validated tool should be a high priority so as to link patients to appropriate services.

## Introduction

Depression is associated with chronic physical illnesses and negatively affects health outcomes [1, 2]. Few affected people access appropriate treatment as depression is often undiagnosed. Diabetes mellitus is a common chronic disease, and in Malawi, the prevalence of raised fasting blood glucose or being currently on medication for diabetes based on the 2009 STEPS survey was 5.6% [3]. A systematic review shows that the prevalence of depression in patients with diabetes mellitus ranges between 6 and 43% [4]. The wide range of reported prevalence estimates may be due to differences in assessment tools and variation in the types of patients in the studies cohorts. Depression in patients with diabetes is often associated with poor glycaemic control, poor adherence to medication, and rapid development of complications [5–7]. For instance a diabetic complication such as diabetic neuropathy is associated with reduced quality of life, poor sleep, depression and anxiety [8]. Comorbid depression is associated with a decrease in metabolic control, poor adherence to medication and diet regimens, a reduction in quality of life, and an increase in health care expenditures. Consequently, poor metabolic control may exacerbate depression and diminish response to depression treatment [9]. Evidence indicates that depression complicate diabetes management, increase the length of hospital stays, and almost doubled the cost of diabetes management [10]. Literature also suggests that duration of diabetes influences depressive symptoms [11]. Furthermore, depression is often undiagnosed and untreated in this patient population [12–16]. Despite the high prevalence and its impact on diabetes, the potential for depression treatment to improve diabetes care outcomes has received little attention in both low and middle income countries (LMIC) [17–19], and high income countries. In LMIC this lack of attention is partly because of limited capacity in depression management and the absence of any routine practice to identify and manage depression in NCD care.

Malawi is facing a growing challenge of non-communicable diseases (NCD), including diabetes mellitus [20–23]. Furthermore, Malawi has a large treatment gap for common mental disorders including depression with a limited number of mental health professionals serving a population of > 18 million. Prevalence of depression between 19 and 30% has been reported among adult primary care attendees in Malawi [24, 25]; none of these cases of depression were identified by clinicians in routine care [24, 25]. There are no published data regarding the prevalence of depression among patients with diabetes attending NCD clinics, or of levels of detection by clinic staff. Currently, there is no routine screening for depression in the NCD clinics in the country.

Therefore, the aim of this study was to determine the prevalence of major depression and its detection by clinicians among adult type 2 diabetes mellitus (T2DM) patients attending NCD clinics in Lilongwe, Malawi.

## Materials and methods

### Setting and participants

We carried out a cross-sectional study from December 2017 till April 2018 among patients suffering from diabetes mellitus. We conducted the study at two diabetes clinics in two facilities within Lilongwe district. The catchment population of the two clinics and the staffing levels have been described elsewhere [26]. The patients attending to the clinics are not routinely screened for any mental health problems. We included adult patients with T2DM who provided consent to participate in the study. The exclusion criteria included patients with Type 1 diabetes mellitus, patients who were too ill, and those suffering from an illness causing inability to communicate.

### Data collection

We collected data from consecutive adult participants with T2DM attending the diabetes clinic at the two facilities. We chose a consecutive sampling technique because it includes all patients who are accessible within the defined study time period [27]. Participants were recruited after they had seen an NCD clinician for routine care. Participants provided written consent after which socio-demographic data was collected and were then screened using the Patient Health Questionnaire-9 (PHQ-9) [28] by a research assistant. The PHQ-9 is a 9-item screening tool which is widely used in clinical practice and research. The PHQ-9 Chichewa version has been validated in Malawi and described elsewhere [29]. All participants who underwent PHQ-9 screening were requested to meet the Structured Clinical Interview for DSM-IV (SCID) [30] interviewer after their clinical consultation. The participants later completed the diagnostic interview administered by the SCID interviewer who was masked from the PHQ-9 scores of the participants. The SCID was used to make a diagnosis of depression among the patients.

The clinicians working at the two facilities' NCD clinics were all clinical officers (COs). Clinical officers are para-medicals who are trained to provide general medico-surgical care; they are trained for four years consisting of 3 years’ college education followed by a year of internship [31–34]. The clinicians in this study had basic mental health training as part of their clinical officer’s training and were not given any extra training for this study. The clinicians reviewed the patients and made entries in the patient's medical booklet (commonly known as a health passport [35]) as well as NCD master-cards (chronic care medical charts which are kept at the clinic). They recorded the presenting complaint, history of the presenting illness, any pertinent past history, findings of the physical examination, diagnosis, any investigations requested and treatment provided. To assess whether depression had been detected as part of routine clinical care, data on the consultation written by the clinician was extracted from the health passports, to ascertain whether a diagnosis of depression was made or not during the clinical encounter.

### Data analysis

For screening data obtained using the PHQ-9, the presence of depressive symptoms was defined as having a total score of at least 5 (PHQ-9 total score ≥ 5). PHQ-9 total scores were categorised as follows: [36, 37].

- 0–4No or minimal depressive symptoms
- 5–9Mild depressive symptoms
- 10–19Moderate depressive symptoms
- 20–27Severe depressive symptoms

DSM IV diagnoses obtained using the SCID were: no depression, minor depression and major depression. Sociodemographic factors measured were: age, sex, education, employment status and marital status. We analysed the association between presence of major depression and sociodemographic factors using logistic regression. Statistical analysis was carried out using both SPSS version 20.0 and Stata 14.

### Ethical approval

The University of Malawi College of Medicine Research and Ethics Committee (COMREC) approved the study protocol. We obtained written informed consent from every participant and fingerprint impressions were taken from consenting participants who could not write. Privacy was maintained during the interviews with participants at the health facilities as the interviews were conducted in a room which provided both visual and audio privacy.

## Results

### Demographic data

Our study included all 323 participants with type 2 diabetes mellitus who were approached, consented and completed the questionnaires (PHQ-9 and SCID); there were no refusals. 244 (76%) were female. Participants’ ages ranged from 21–79 years with a mean (standard deviation) of 54.0 (11.4). 8.4% of the participants were illiterate. Table 1 shows patients’ characteristics.

**Table 1:** Socio-demographic characteristics of study participants

Variable	Frequency (total n = 323)	Percent
Age (years)		
21–30	10	3.1
31–40	33	10.2
41–50	75	23.2
51–60	106	32.8
61–70	85	26.3
71–80	14	4.3
Education		
No education	27	8.4
Primary	153	47.4
Secondary/tertiary	143	44.3
Employment status		
Employed	143	44.3
Unemployed	180	55.7
Gender		
Male	79	24.5
Female	244	75.5
Marital status		
Married	249	77.1
Unmarried	74	22.9

### Severity score of depressive symptoms and prevalence of probable depression based on PHQ-9

The distribution of severity of depressive symptoms based on the PHQ-9 scores is shown in Table 2.

**Table 2:** Severity score of depressive symptoms based on PHQ-9

Severity score	PHQ-9 score	Frequency (total n = 323)	Percent
No or minimal depressive symptoms	0–4	132	40.9
Mild depressive symptoms	5–9	111	34.4
Moderate depressive symptoms	10–19	73	22.6
Severe depressive symptoms	20–27	7	2.2

### Prevalence of depression based on SCID

The prevalence of DSM IV depression diagnoses based on the SCID were: no depression 58.2% (n = 190), minor depression 23.8% (n = 75) and major depression 18% (n = 58).

### Distribution by major depression status and socio-demographic characteristics based on multiple logistic regression analyses

Major depression was found not to be significantly associated with any of the sociodemographic variables: education level (*p* < 0.07) age, gender (*p* < 0.47), marital status *(p* < 0.92), and current employment status (*p* < 0.34) (Table 3).

**Table 3:** Prevalence of major depression by individual socio-demographic characteristics in adults with type 2 diabetes mellitus based on multiple logistic regression analyses

Variable	No Depression		Depression		P-value	OR	95% CI	
	n	%	n	%				
Age (years)								
21–30*	9	90	1	10		*1 (ref)*		
31–40	26	78.8	7	21.2	0.442	2.414	0.255	22.88
41–50	61	81.3	14	18.7	0.617	1.744	0.191	15.42
51–60	87	82.1	19	17.9	0.667	1.604	0.187	13.75
61–70	71	83.5	14	16.5	0.762	1.396	0.167	12.14
71–80	11	78.6	3	21.4	0.624	1.849	0.158	21.63
Education								
No education*	19	70.4	8	29.6		*1 (ref)*		
Formal education	246	83.1	50	16.9	0.069	1.082	0.404	1.035
Employment status								
Employed*	122	85.3	21	14.7		*1 (ref)*		
Unemployed	143	79.4	37	20.6	0.340	1.082	0.920	1.272
Gender								
Male*	68	86.1	11	13.9		*1 (ref)*		
Female	197	80.7	47	19.3	0.466	1.315	0.629	2.746
Marital status								
Married*	204	81.9	45	18.1		*1 (ref)*		
Unmarried	61	82.4	13	17.6	0.922	0.966	0.481	1.937

* Reference category

### Detection of depression by NCD Clinicians

None of the participants were diagnosed as having depression during routine clinical assessment by the NCD clinicians. This included the 58 participants who were identified as having major or minor depression using SCID.

## Discussion

Recognising a mental disorder such as depression in patients with physical illness is important in clinical management and improving patients’ clinical outcomes. In this population of adult type 2 diabetes mellitus (T2DM) patients attending non communicable diseases (NCD) clinics in Lilongwe, Malawi, we investigated the prevalence and rate of routine detection of depression, finding that 18% of participants met criteria for major depression, none of whom were detected in routine practice by the NCD clinicians.

### Prevalence of depression among people with T2DM

Evidence indicate that 18%–25% of people with T2DM will meet DSM criteria for a major depressive episode using the SCID [11]. The prevalence of major depression in T2DM in our study is comparable to prevalence estimates of 19% and 30% found in two studies of general primary care outpatient attendees in Malawi [24, 25]. There is some overlap with the populations in the previous studies as these were conducted in facilities that did not have dedicated NCD clinics, so will have included patients with T2DM. The finding of our study that one-fifth of patients with T2DM (18%) suffered from major depression is similar to the findings of a systematic review by Roy and Lloyd where the they found a prevalence of 19.1% (range 6.5–33%) [4] however the finding is considerably higher compared to that of a collaborative study carried out in 14 countries which found a prevalence of 10.6% [38]. The rate of major depression in this study is also comparable to rates of depression in studies from Nigeria with a prevalence of 19.4% based on the SCID [39] and from Ethiopia with a prevalence of 17% on the Beck Depression Inventory (BDI) [40].

In contrast to our study, several studies in other African countries have shown higher prevalence of depression among T2DM patients. A study in Morocco found that patients with T2DM had higher prevalence of major depression (33.1%) [41] while studies in South Africa found and in Egypt found 46% and 32% respectively [39]. Similarly a systematic review conducted in Ethiopia showed that the prevalence of depression among T2DM patients was 39.73% [42]. In other LMICs the findings of higher prevalence of major depression are also similar. For instance a multi-centre study conducted in Pakistan found that 43.5% of T2DM patients had depression [43]. A Mexican study also found a higher prevalence of 48.27% [44] compared to our study.

### Depression screening and diagnosis

Depression is diagnosed using a structured clinical interview, such as the SCID and other interviews such as Composite International Diagnostic Interview (CIDI). However the interviews vary in terms of length as such they can be time-consuming therefore depression can be screened accurately using a few questions such as the PHQ-9 [11]. A variety of screening tools for depression are available, however few have been developed for use in LMIC settings [45]. In our study, the majority of patients (34.4%) had mild depression, 22.6% had moderate and 2.2% had severe depression based on PHQ-9 scores. Our finding is similar to findings in other LMICs, for instance a study in Iran that showed that 38% of patients had mild depression, 30% had moderate depression, and 13% had severe depression on the BDI [46]. Further, the findings are also similar to a study in Tanzania which showed that 22.1% had mild depression, and 8.2% had moderate depression while none had severe depression based on the PHQ-9 [6]. An Indian study also found almost similar finding which showed that 25% had mild depression, 12.5% had moderate depression, and 1.3% had severe depression on the Major Depression Inventory (MDI) [7]. In contrast to our study, a Bangladesh study found that majority of patients (20.2%) had severe depression rather than mild to moderate depression (14.6%) using the Centre for Epidemiological Studies Depression Scale [47]. One of the reasons could be because of the relatively small size of the data sample in the Bangladesh study compared to our study and the other reason could be the use of different tools.

### Interventions for comorbid depression and diabetes

Most low and middle income countries have a large treatment gap for common mental disorders including depression [48]. Studies suggest that one way of reducing the treatment gap is the integration of mental health into other health care services [49, 50]. Integrating depression screening and treatment into primary care to improve access to mental health services mostly in low- and middle-income countries (LMICs) is being advocated [51]. Furthermore, it has been suggested that mental health care and NCD care should be offered together in primary care platforms due to the growing burden of both [52]. There is evidence that demonstrates the effectiveness of a range of mental disorder interventions, including medicines, psychological treatments, and social interventions delivered by non-mental health specialist in LMICs [53]. A systematic review of collaborative care for patients with comorbid conditions provides evidence to support the effectiveness of integrated care in improving depression outcomes and improved adherence to treatment for both depression and diabetes [54].

Despite the advocacy of the initiative, many barriers to integration exist. For instance, a potential barrier to integrating screening into routine practice and ensuring patients with depression receive the necessary required care is that many health workers in LMICs are not trained or do not have time to screen for depression when seeing patients [45].

Despite the high prevalence of depression, none of the patients who were diagnosed with major depression by the research team using the SCID were detected and diagnosed as such by the NCD clinician. This finding agrees with other studies conducted among patients with T2DM [12–16]. The reasons for non-detection of depression in this setting could be due to lack of knowledge and skills, lack of integration of mental health in NCD care and lack of routine screening for depression. These reasons are in line with the results from general primary care clinics studies in Malawi [24, 25]. The failure to detect and manage depression will affect management of T2DM as evidenced in other studies [15, 16]. Several studies have demonstrated that depression treatment effectively reduces the severity of depression in patients with diabetes and also produces better glycaemic control [55], significantly improve HbA1C [56] restore mental health and improve medical outcome [57]. As such if depression in patients with diabetes is not recognized, it will go untreated and consequently the medical and mental health outcomes will be negatively affected. Detecting depression in the clinical setting is aided by validated tools that can be easily administered with limited resources. Improved detection of depression through the use of the PHQ-9 by the clinicians would lead to the initiation of treatment or referral to mental health services for treatment which may consequently lead to improved outcomes and lower complications. Therefore, NCD clinicians have to be equipped with the necessary knowledge and skills in detection and treatment.

Based on multivariable logistic regression, the sociodemographic factors including age, gender, education, marital status and employment were not associated with depression. Related studies have gotten varying results with many similarly finding no association with this set of factors [1, 17, 58–63]; but, some individual studies showing associations with them [64–67]. In the present study, three quarters of the participants were females. However, it is unlikely that this had an influence on the results for females as there was no evidence of a difference in the odds of depression between the two genders. The over representation of females in our study is similar to other studies like the Malawi STEPS Survey [68], which may be due to the increased health care seeking behaviour by females relative to males.

One notable strength of this study is that it used a gold standard, the SCID for depression that had previously been used in different studies in Malawi. Furthermore, the quality of administration of the PHQ-9 and of the SCID was very good as the research assistants had training in administration of the research tools and received regular supervision from the principal investigator. To our knowledge, this is the first study analyzing depression among patients with diabetes in Malawian population.

### Study limitations

The results of this study should however be interpreted in light of limitation. The limitation of this study is that the participants were selected through a consecutive sampling technique from two specialized NCD clinics in Lilongwe which may not be representative of the wider population. However, despite this limitation, the paper fills an important knowledge gap regarding the prevalence and detection of depression among patients with diabetes in Malawi, given the dearth of evidence in LMIC settings. In this regard, the paper shows a crucial gap and has the potential to stimulate policy makers and clinicians to develop interventions to improve detection of depression among patients with diabetes.

## Conclusions

Depression is prevalent among patients with type 2 diabetes mellitus attending NCD clinics in Malawi. The evidence from this study shows that depression is undiagnosed in patients with diabetes in NCD clinics. Clinicians in NCD clinics must know how to screen for depression and ensure availability of treatment. Clinicians should consider using screening tools to identify depression among patients with type 2 diabetes. Furthermore, there must be a parallel focus on establishing mental health services to which these identified patients could be connected. It is therefore important to explore ways to identify depression in NCD clinics.

## Data Availability

The datasets used and/or analyzed during the current study are available from the corresponding author on reasonable request.
